# Effectiveness of Naturally Acquired and Vaccine-Induced Immune Responses to SARS-CoV-2 Mu Variant

**DOI:** 10.3201/eid2808.220584

**Published:** 2022-08

**Authors:** Edmilson F. de Oliveira-Filho, Bladimiro Rincon-Orozco, Natalia Jones-Cifuentes, Brigitte Peña-López, Barbara Mühlemann, Christian Drosten, Andres Moreira-Soto, Jan Felix Drexler

**Affiliations:** Charité-Universitätsmedizin Berlin, corporate member of Freie Universität Berlin and Humboldt-Universität zu Berlin, Institute of Virology, Berlin, Germany (E.F. de Oliveira-Filho, B. Mühlemann, C. Drosten, A. Moreira-Soto, J.F. Drexler);; Universidad Industrial de Santander School of Medicine, Bucaramanga, Colombia (B. Rincon-Orozco, N. Jones-Cifuentes, B. Peña-López);; German Centre for Infection Research, Berlin (B. Mühlemann, C. Drosten, J.F. Drexler)

**Keywords:** COVID-19, SARS-CoV-2, coronavirus disease, immune evasion, acquired immunity, neutralization, vaccine efficacy, virus variants, severe acute respiratory syndrome coronavirus, viruses, Colombia

## Abstract

SARS-CoV-2 Mu variant emerged in Colombia in 2021 and spread globally. In 49 serum samples from vaccinees and COVID-19 survivors in Colombia, neutralization was significantly lower (p<0.0001) for Mu than a parental strain and variants of concern. Only the Omicron variant of concern demonstrated higher immune evasion.

Diverse SARS-CoV-2 variants have arisen during the pandemic. As of May 4, 2022, there had been 2 recognized variants of concern (VOC), Delta and Omicron, in addition to earlier emerging VOCs Alpha, Beta, and Gamma and strains previously categorized as variants of interest (VOI). Many VOIs have been understudied in terms of pathogenesis, transmissibility, and potential for immune escape. Delta and Omicron illustrate how variants emerging in tropical settings can spread globally.

Mu was first reported as a VOI in early January 2021 in northern Colombia. While outcompeting other locally circulating variants, Mu spread to additional countries, such as Ecuador, United States, Mexico, and Spain; as of early 2022, it was still circulating at low levels in Colombia (*1*). Mu caused 70% of all COVID-19 cases in Colombia during May–July 2021 (Figure 1), a period which also accounted for the highest number of deaths in Colombia during the pandemic, suggesting substantial pathogenicity of Mu (*1*). Mu was later outcompeted by Delta and Omicron, and the number of Mu-related cases gradually decreased through the end of 2021 (Figure 1).

**Figure 1:**
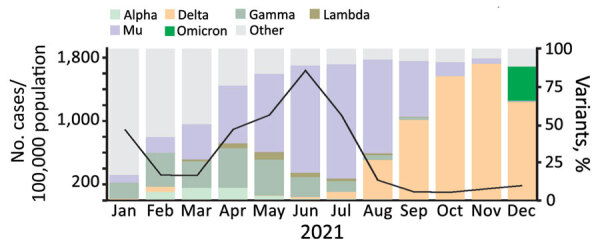
Incidence of SARS-CoV-2 and circulation of variants, by month, Colombia, 2021. Data on variant circulation was obtained from GISAID (https://www.gisaid.org) and data on the number of cases in Colombia from the Our World in Data database (https://www.ourworldindata.org).

Recent studies relying on data from spike-based pseudovirus testing suggested substantially lower neutralization of Mu compared with the parental B.1 virus in antiserum samples from persons in Japan and China who had received either the BNT162b2 (Pfizer-BioNTech, https://www.pfizer.com) or Sinovac (http://www.sinovac.com) vaccines or recovered from COVID-19 (*2*,*3*). Because of inherent limitations in pseudovirus-based systems for reproducing response variations based on natural infection (*4*), regional differences of immune responses (*5*), and different vaccines used in Colombia, we comparatively characterized the neutralization of Mu and VOCs using fully infectious viruses and serum samples from persons in Colombia. The study was approved by the Ethics Committee of the Universidad Industrial de Santander (protocol 4110) and by the Ethics Committee of the Charité-Universitätsmedizin Berlin (protocol EA2/031/22). All participants provided written informed consent.

## The Study

By March 2022, ≈68% of the population of Colombia had been vaccinated, predominantly with spike-based mRNA (BNT162b2), vectored (AZD1222; AstraZeneca, https://www.astrazeneca.com), and chemically inactivated whole virus–based vaccines (CoronaVac) (Appendix Figure 1). To investigate the potency of natural and vaccine-derived immunity, we tested and compared the neutralization activity in 49 serum samples from vaccinated and naturally infected persons in Colombia. Among vaccinated persons, we tested serum from 32 persons sampled in October 2021. Of those, 10 vaccinated with BioNTech-Pfizer were tested a median 99.5 d (range 65–170) after completing vaccination, 7 vaccinated with AstraZeneca were tested a median 146.0 d (range 129–173) after completing vaccination, and 15 vaccinated with CoronaVac were tested a median 46.0 d (range 28–131) after completing vaccination. We tested serum samples from 17 persons who tested positive for SARS-CoV-2 antibodies (MAGLUMI 2019-nCoV IgG; Snibe Diagnostic, https://www.snibe.com) (Table 1; Appendix Table 1) during a seroprevalence study conducted in November 2020. To control whether persons vaccinated with spike-based vaccines were not previously infected, serum samples were tested against the SARS-CoV-2 IgG nucleocapsid protein by ELISA (SARS-CoV-2 NCP kit; Euroimmun, https://www.euroimmun.com) (Table 2). We used 50% plaque reduction neutralization tests to obtain neutralizing titers against an early isolate and the Alpha, Beta, Delta, Gamma, Omicron BA.1, and Mu variants (Appendix).

**Table 1:** Median age and days after the second dose of vaccinated persons, by vaccine type, at time of sampling among persons in Colombia*

Vaccine groups	Days after second dose (range)	Age, y (range)
AstraZeneca	146 (129–173)	66.0 (61–72)
Pfizer-BioNTech	99.5 (65–170)	44.6 (27–65)
Sinovac	46.0 (23–131)	44.5 (23–92)

*AstraZeneca (AZD1222), https://www.astrazeneca.com; Pfizer-BioNTech (BNT162b2), https://www.pfizer.com; Sinovac (CoronaVac), http://www.sinovac.com.

**Table 2:** ELISA results and endpoint titers for vaccinee and naturally infected individual serum samples from persons in Colombia*

Group	Patient ID	Nucleocapsid IgG ELISA†	Neutralizing titer by PRNT_50_						
			WT	Mu	Alpha	Beta	Gamma	Delta	Omicron
AstraZeneca	AZ2	0.15	204	41	154	79	64	77	13
	AZ3	0.07	453	123	381	305	306	470	25
	AZ4	0.11	75	3	91	16	20	24	6
	AZ5	0.12	76	9	104	3	13	29	2
	AZ6	0.13	34	4	45	3	23	15	0
	AZ9	0.08	179	35	189	75	128	84	10
	AZ10	0.07	319	9	153	47	55	26	8
Pfizer-BioNTech	PF1	0.14	119	9	85	1	18	38	3
	PF2	0.04	28	3	43	35	15	17	3
	PF3	0.15	262	62	158	130	101	149	19
	PF4	0.06	754	121	715	204	226	187	43
	PF5	0.05	501	87	320	48	91	259	9
	PF6	0.07	123	10	119	52	15	11	3
	PF7	0.19	214	9	70	5	0	125	3
	PF8	0.05	207	18	167	28	25	66	3
	PF9	0.09	715	10	273	0	46	108	2
	PF10	0.62	1043	132	1036	343	333	799	47
Sinovac	SVN1	2.96	51	54	72	36	66	83	0
	SVN2	1.68	47	9	24	23	21	22	0
	SVN3	0.46	41	6	1	1	18	1	0
	SVN4	2.43	118	61	151	111	89	87	25
	SVN7	0.97	363	162	347	407	188	259	56
	SVN8	0.81	303	5	93	26	30	61	5
	SVN9	0.69	53	0	32	3	15	35	0
	SVN10	1.61	65	4	27	0	10	66	1
	SVN12	0.29	52	8	52	1	20	21	0
	SVN13	0.39	387	24	126	126	35	130	7
	SVN15	2.81	145	175	168	197	147	133	19
	SVN16	0.40	67	2	6	25	10	21	3
	SVN17	0.07	24	1	1	5	0	15	3
	SVN18	0.37	65	0	3	4	0	24	7
	SVN20	1.88	686	464	612	155	131	503	16
Naturally infected	EA210	ND	696	146	825	595	167	177	2
	EA234	ND	142	4	86	83	67	9	0
	EA238	ND	1,080	48	1080	314	541	79	5
	EA245	ND	70	2	61	154	44	0	0
	EA332	ND	93	10	43	94	1	20	0
	EA334	ND	140	6	74	115	7	16	3
	EA340	ND	77	2	24	61	0	14	2
	EA352	ND	1,080	113	1,080	578	870	59	3
	EA354	ND	336	119	423	972	90	628	0
	EA380	ND	918	43	281	630	151	63	17
	EA396	ND	139	24	98	88	14	21	0
	EA413	ND	1,080	18	864	1,080	260	17	11
	EA422	ND	2	20	28	6	123	100	0
	EA439	ND	398	171	283	812	79	62	9
	EA485	ND	357	87	531	206	114	14	0
	EA501	ND	17	13	86	80	1	0	2
	EA520	ND	166	36	154	211	16	141	1

*AstraZeneca (AZD1222), https://www.astrazeneca.com; Pfizer-BioNTech (BNT162b2), https://www.pfizer.com; Sinovac (CoronaVac), http://www.sinovac.com. ND, not determined; PRNT_50_, 50% plaque reduction neutralization test; WT, wild-type. †Cut-off ≥0.8 was considered positive.

**Figure 2:**
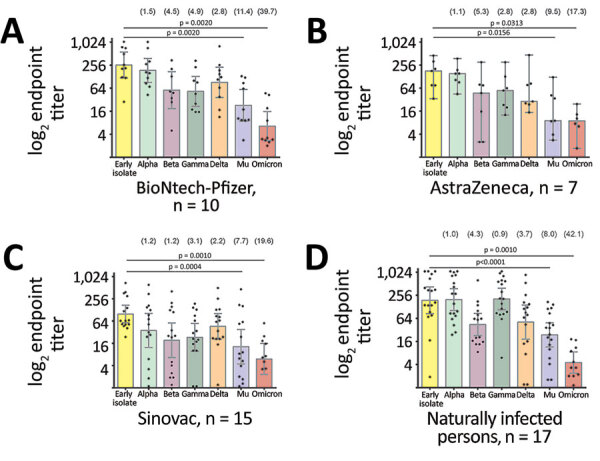
Comparative neutralization of the Mu SARS-CoV-2 variant in Colombia. A–C) Neutralization of SARS-CoV-2 variants from serum samples from persons fully immunized with BNT162b2 (Pfizer-BioNTech, https://www.pfizer.com) (A), AZD1222 (AstraZeneca, https://www.astrazeneca.com) (B), or CoronaVac (Sinovac, http://www.sinovac.com) (C). D) Neutralization of SARS-CoV-2 variants by serum samples from naturally infected persons who tested positive for SARS-CoV-2 antibodies during a seroprevalence study in November 2020. For all panels, each point represents the reciprocal plaque reduction neutralization test endpoint titer of 1 tested serum sample for different SARS-CoV-2 variants; colored bars indicate geometric mean titers, and error bars represent 95% CIs. Values in parentheses above bars represent reduction compared to the parental strain. Statistical significance was determined by the Wilcoxon matched signed-rank test; p values are indicated. For clarity of presentation, only significant values between the early isolate and the Mu variant are shown.

Neutralizing antibody titers against Mu were significantly lower than those against the parental isolate (p<0.0001 by Wilcoxon matched-pairs signed-rank test) in all serum samples tested in this study, irrespective of whether immune responses were elicited by vaccination or by natural infection. Vaccine-derived antibodies neutralized Mu on average 8.1-fold (p<0.0001 by Wilcoxon test) less than the parental strain resembling the vaccine backbones (Figure 2, panels A–C; Appendix Figure 2). We found a similar 8.0-fold reduced neutralization of Mu (p<0.0001 by Wilcoxon test) for the group of naturally infected persons (Figure 2, panel D). Despite the relatively lower neutralization potency observed in serum samples from persons immunized with the inactivated full virus-based vaccine Sinovac, observed differences in the ability to neutralize Mu compared with the parental strain among the 3 vaccine groups were not statistically significant (range 7.7–11.4-fold; p = 0.8298 by Kruskal-Wallis test) (Figure 2).

Compared with other variants, neutralizing antibody titers from serum samples of both naturally infected persons and vaccinees were lower against Mu than against all VOCs except for Omicron (Figure 2, panels A and B). Therefore, our results provide strong evidence for immune evasion of the Mu VOI on the basis of results from robust neutralization testing using full viral isolates. Neutralization of Mu by vaccine-induced antibodies was significantly lower than for Beta (p = 0.0083 by Wilcoxon text), for which immune evasion properties led to the suspension of AstraZeneca usage in South Africa (*6*), and Gamma, which resulted in breakthrough infections in Latin America (*7*). Immune evasion of Mu is consistent with shared mutations in spike protein residues associated with immune evasion in Beta and Gamma, such as E484K (*8*). In addition, the mutation leading to the amino acid exchange R346K in Mu is known to be involved in the evasion of monoclonal antibody–mediated neutralization (*9*), and genomic exchanges occurring at 3 adjacent sites (Y144T, Y145S, and insertion of the amino acid asparagine [N] between spike residues 145 and 146) have been associated with the immune escape properties of Mu (*10*,*11*).

Antigenic cartography was recently employed to map the antigenic relationship between the SARS-CoV-2 Omicron and Delta VOCs and other previously circulating VOCs and VOIs (S.H. Wilks et al., unpub. data, https://www.biorxiv.org/content/10.1101/2022.01.28.477987v1). Among the serum samples from Colombia vaccinees, there was a high antigenic distance between Mu and most variants from other serum samples, which clustered together with the parental strain and Alpha (Appendix Figure 3). Of note, antibody responses in naturally infected persons supported past infection with strains bearing similarities to early SARS-CoV-2 isolates and the Gamma variant (Figure 2, panel D). Antibody reactivity in naturally infected persons was thus in concordance with the circulation of SARS-CoV-2 variants in South America during the time of sampling in late 2020 (*12*), supporting the robustness of our data.

Our study was limited by different time points for sampling of vaccinees and the lack of information on natural infections altering immune responses in vaccinees. However, lack of detectable N-protein antibody responses and the absence of clinical records suggestive of COVID-19 infection in vaccinees immunized with spike-based vaccines supports the robustness of our data despite the vaccinees’ unclear infection histories.

## Conclusions

Our data highlight the importance of continuous monitoring for the emergence of new SARS-CoV-2 variants and strains and the timely identification of those variants with potential to evade naturally elicited and vaccine-derived immune responses, using local sampling specimens in the context of regional epidemiologic conditions. Moreover, our data confirmed the potential of Mu to partially evade immune responses, which may affect the efficacy of vaccination programs in southern America and other areas (*7*,*13*). Further studies are warranted to evaluate the pathogenicity of and cell-mediated immunity against Mu and the ability of immune responses associated with Mu to neutralize other SARS-CoV-2 variants. However, because vaccination boosters still provide some degree of protection against severe disease from Omicron (*3*,*14*), which shows more immunity evasion than Mu, vaccination will likely still provide protection against severe disease from Mu.

Appendix Additional information on robustness of SARS-CoV-2 Mu variant against naturally acquired and vaccine-induced immune responses among persons in Colombia.
