# Supplementary material for: Effectiveness of Naturally Acquired and Vaccine-Induced Immune Responses to SARS-CoV-2 Mu Variant
Source: Emerg Infect Dis. 2022 Aug;28(8):1708–12. doi: 10.3201/eid2808.220584 (PMC9328909; doi:10.3201/eid2808.220584)
Supplement: Appendix — Additional information on robustness of SARS-CoV-2 Mu variant against naturally acquired and vaccine-induced immune responses among persons in Colombia. [file 22-0584-Techapp-s1.pdf]

Article DOI: https://doi.org/10.3201/eid2808.220584

Effectiveness of Naturally Acquired and Vaccine-Induced Immune Responses to SARS-CoV-2 Mu Variant

Appendix

Participant Recruitment and Sampling

The study was approved by the Ethics Committee of the Universidad Industrial de Santander (protocol 4110) and by the Ethics Committee of the Charité Universitätsmedizin-Berlin (Protocol EA2/031/22). All patients provided written informed consent. To control whether the vaccinated persons also had been naturally infected, subjects were followed up and did not report clinical symptoms or direct contact with persons who tested positive until sampling. In addition to that, all persons vaccinated with the spike-based vaccines from Pfizer and AstraZeneca tested negative for antibodies against the SARS-CoV-2 N protein, suggesting lack of natural infection and consistent with recording of clinical symptoms.

50% Plaque Reduction Neutralization Tests

We used a parental SARS-CoV-2 B.1 lineage strain (Pango version 3.1.17) sampled in January 2020 (Munich/ChVir929/2020 strain, GISAID accession: EPI_ISL_406862), containing one mutation (D614G) in the spike-encoding gene only compared to the SARS-CoV-2 reference sequence used for vaccine production (Isolate Wuhan-Hu-1 GenBank accession number: NC045512). We used the following SARS-CoV-2 variants: Alpha (ChVir21652/2020, GISAID accession: EPI_ISL_802995), Beta (ChVir22131/2021, GISAID accession: EPI_ISL_862149), Gamma (NH-RIVM_10915/2021, GISAID accession: EPI_ISL_943045), Delta (454236/2021, GISAID accession: EPI_ISL_4566914), Mu (H3/2021, GISAID accession: EPI_ISL_6665693) and Omicron (hCoV-19/Netherlands/NH-RIVM-71076/2021, GISAID accession: EPI_ISL_6841611.2; Pango lineage: BA.1.17.2). A total of 60 plaque-forming units were incubated with serum dilutions of 1:40, 1:120,1:360, and 1:1080 for 1 h, and afterwards added onto a monolayer containing 1.8×10^5 Vero E6 cells per well in a 12-well plate. After 1 h of

incubation, an overlay containing DMEM with 1% FCS and 2% Avicell was added, and cells were further incubated for 3 d for Mu and 2 d for the other variants. The overlay medium was removed, and cells were fixated with 6% paraformaldehyde and stained with crystal violet. PRNT50endpoint titers were calculated using a logistic regression function in GraphPad prism6 (www.graphpad.com).

Antigenic Cartography

Antigenic cartography was done using the R package Racmacs (onhttps://acorg.github.io/Racmacs) as described elsewhere (S.H. Wilks; unpub. data, https://www.biorxiv.org/content/10.1101/2022.01.28.477987v1). Comparative neutralization of SARS-CoV-2 variants by serum samples from persons fully immunized with the different vaccines (BioNTech-Pfizer BNT162b2, AstraZeneca AZD1222, and CoronaVac). Each point represents 50% plaque reduction neutralization test endpoint titers of 1 tested serum using different SARS-CoV-2 variants.

Appendix Table. List of samples and reciprocal $PRNT_{50}$ endpoint titers of serum samples for vaccinated persons*†

Sample ID	Early isolate	Mu	Alpha	Beta	Gamma	Delta	Omicron	D after 2nd dose	Age	Vaccine
AZ2	204	41	154	79	64	77	13	129	64	AstraZeneca
AZ3	453	123	381	305	306	470	25	173	69	AstraZeneca
AZ4	75	3	91	16	20	24	6	173	72	AstraZeneca
AZ5	76	9	104	3	13	29	2	146	61	AstraZeneca
AZ6	34	4	45	3	23	15	0	165	72	AstraZeneca
AZ9	179	35	189	75	128	84	10	142	61	AstraZeneca
AZ10	319	9	153	47	55	26	8	142	63	AstraZeneca
PF1	119	9	85	1	18	38	3	113	65	BioNTech
PF2	28	3	43	35	15	17	3	170	42	BioNTech
PF3	262	62	158	130	101	149	19	110	46	BioNTech
PF4	754	121	715	204	226	187	43	110	27	BioNTech
PF5	501	87	320	48	91	259	9	121	29	BioNTech
PF6	123	10	119	52	15	11	3	80	46	BioNTech
PF7	214	9	70	5	0	125	3	88	54	BioNTech
PF8	207	18	167	28	25	66	3	66	35	BioNTech
PF9	715	10	273	0	46	108	2	65	49	BioNTech
PF10	1043	132	1,036	343	333	799	47	89	53	BioNTech
SVN1	51	54	72	36	66	83	0	89	56	CoronaVac
SVN2	47	9	24	23	21	22	0	33	23	CoronaVac
SVN3	41	6	1	1	18	1	0	31	30	CoronaVac
SVN4	118	61	151	111	89	87	25	27	28	CoronaVac
SVN7	363	162	347	407	188	259	56	37	27	CoronaVac
SVN8	303	5	93	26	30	61	5	37	54	CoronaVac
SVN9	53	0	32	3	15	35	0	41	25	CoronaVac
SVN10	65	4	27	0	10	66	1	46	26	CoronaVac
SVN12	52	8	52	1	20	21	0	105	60	CoronaVac
SVN13	387	24	126	126	35	130	7	90	57	CoronaVac
SVN15	145	175	168	197	147	133	19	131	92	CoronaVac
SVN16	67	2	6	25	10	21	3	90	54	CoronaVac
SVN17	24	1	1	5	0	15	3	97	56	CoronaVac
SVN18	65	0	3	4	0	24	7	81	52	CoronaVac
SVN20	686	464	612	155	131	503	16	46	28	CoronaVac

*AZ, AstraZeneca; PF, Pfizer; SVN, Sinovac

†Because we followed the Colombian vaccination program, it was not possible to collect samples the same time after completing the recommended vaccination scheme and the subjects' age were variable.

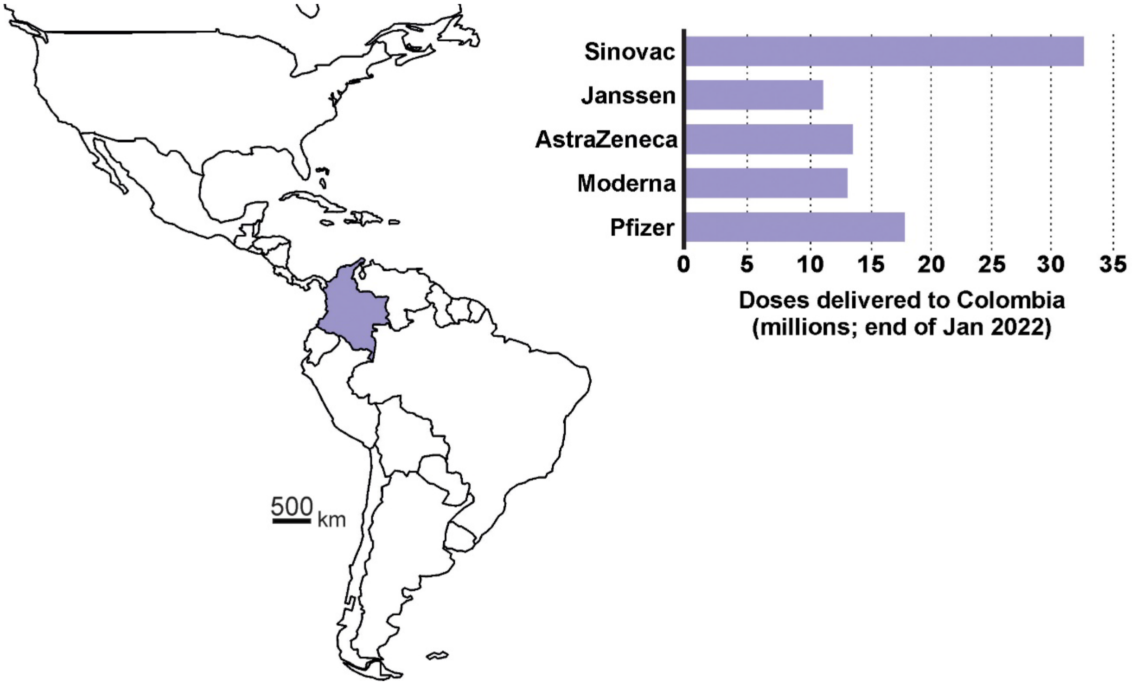

Appendix Figure 1. Vaccines doses delivered to Colombia as of January 2022 (https://www.minsalud.gov.co).

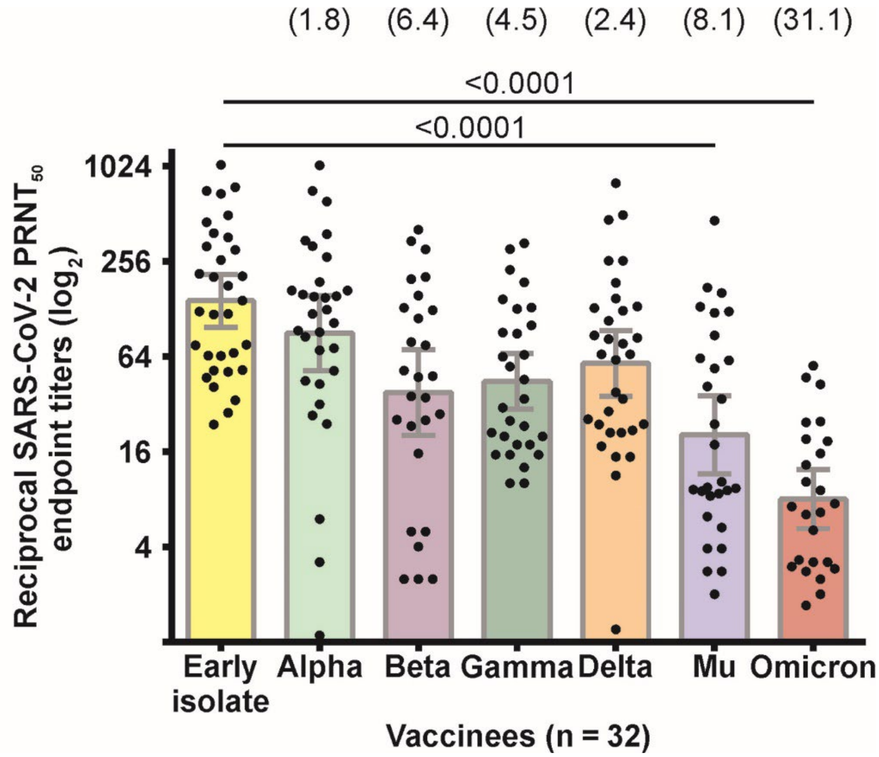

Appendix Figure 2. Comparative neutralization of SARS-CoV-2 variants by serum samples from persons fully immunized with the different vaccines (BioNTech-Pfizer BNT162b2, AstraZeneca AZD1222, and CoronaVac). Each point represents 50% plaque reduction neutralization test endpoint titers of 1 tested serum using different SARS-CoV-2 variants; the bars indicate the geometric mean titers, and the grey error bars represent 95% CI. Statistical significance was determined by the Wilcoxon matched signed-rank test and p-values are indicated on top. For clarity of presentation, only significant values between the early isolate and the Mu variant are shown.

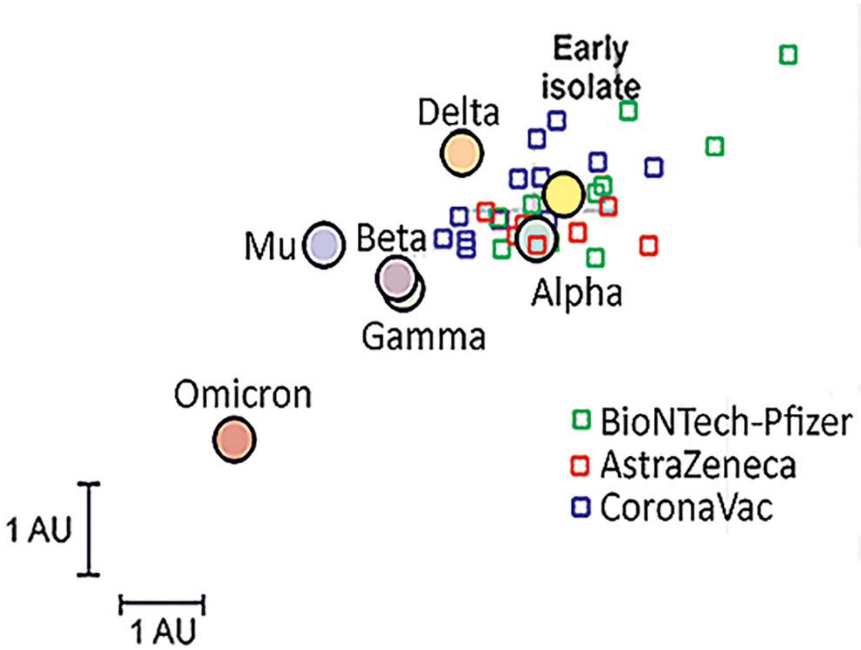

Appendix Figure 3. Antigenic cartography of SARS-CoV-2 variants based on serum samples used in Figure 2 A–C. Each square corresponds to a serum sample tested. The colored circles indicate the tested SARS-CoV-2 variants. One grid square (1 antigenic unit) corresponds to a 2-fold serum dilution in the $PRNT_{50}$ assay. To decrease uncertainty in the antigenic cartography, $PRNT_{50}$ all endpoint titers <10 were considered as exactly <10. Antigenic mapping was not done for naturally infected persons because we could not rule out infection with multiple SARS-CoV-2 variants leading to heterogeneous antibody responses preventing a meaningful antigenic map.
